# Epidemiologic Study on Social Determinants of Health: What’s Next?

**DOI:** 10.2188/jea.JE20150082

**Published:** 2015-07-05

**Authors:** Naoki Kondo

**Affiliations:** Department of Health and Social Behaviour/Department of Health Education and Health Sociology, School of Public Health, The University of Tokyo, Tokyo, Japan

**Keywords:** health inequality, child, social determinants of health, policy

In this issue, Kachi et al have reported novel evidence on health inequalities among young Japanese children and adolescents, showing that overweight is more prevalent among children who are socioeconomically disadvantaged in terms of parental income, expenditure, occupation, and education.^1^ The results are not surprising, as they are similar to data from other parts of the world, but the key message we should take from the results is that Japan—a country long considered an egalitarian society—is not an exception. In its final report, the World Health Organization’s (WHO) Commission on Social Determinants of Health provided three essential policy recommendations.^2^ The first one concerns “improving daily living conditions” by addressing “the well-being of girls and women and the circumstances in which their children are born, put[ting] major emphasis on early child development and education for girls and boys…”. Although we still do not know whether health inequalities among Japanese children have widened or narrowed in recent decades, nor the potential macro-socioeconomic determinants of such trends, epidemiology and public health should go beyond health policy, dealing with wider social environments and policies that determine health. It is time to start managing health inequalities among Japanese children at a higher level.

The WHO report also recommends that we “measure and understand the problem and assess the impact of action”. The analysis by Kachi et al provides a good example of how such measurement or monitoring of health inequality is possible using data created from the individual linkage of multiple governmental surveys. Although the data were reported as an academic finding published in a peer-reviewed journal, this kind of health inequality monitoring should become a routine annual public endeavour, with which any governmental policy reforms can be evaluated from the perspective of health inequalities.^3^ For example, recent policy reforms that may directly and indirectly affect child health, including the establishment of the Child Poverty Act in 2013 and recent modifications of the child allowance policy, can be evaluated.

Nevertheless, the current condition of available secondary data from governmental surveys presents some critical challenges. For example, Kachi et al linked two national surveys to use both health and socioeconomic variables. They linked two datasets using their original individual identification (ID) codes, which were created with information on respondents’ area of residence, sex, age, household identification number, and within-household ordered number of its members. Unfortunately, this approach could suffer from linkage errors due to the existence of multiple same-age household members within a household and incidental incorrect numbering of household members between the two surveys. Other potential limitations include the small sample size and large amounts of missing data, which may make the results less representative.

The solution to these challenges is very simple and remarkably effective: use national personal ID numbers. For example, the forthcoming ‘My Number’ system, which is a new Japanese tax and social security ID system, can be used for epidemiologic research purposes. Nordic states are the global leaders in terms of the application of such identity systems in scientific research; there are many examples that demonstrate that the use of such individual ID-based data linkage is effective in monitoring health inequality over time.^4^

Use of large national datasets is also very important when evaluating health issues among minorities. In Japan, for example, evidence of the health statuses of immigrants, sexual minorities, and people who are extremely poor and socially isolated is scarce because of the difficulty in capturing data on those populations through regular sampling surveys. Reliable healthcare databases (eg, health insurance claims and clinical registries) should also be useful for monitoring health inequality across subpopulations if they are individually linked with external socioeconomic data (eg, national census data). The epidemiologic research community should continue to advocate for the increased availability of such data.

Establishing evidence of the existence of health inequality, however, is far from sufficient for managing health inequality. Further evidence is needed in terms of the differential responses to interventions aiming to reduce health inequality across subpopulations with different demographic and socioeconomic backgrounds. There has been debate that some intervention approaches—for example, the simple provision of cancer screening opportunities and health information campaigns—may actually increase health inequality because, as studies show, poorly educated people are less likely to participate in cancer screening programs, even when they are provided at no cost.^5^ For the sake of causal inference, the best way to determine effective interventions is through conducting randomized trials. Unfortunately, such trials are extremely difficult and costly in the field of social determinants of health. There are some exceptions, however, including the large-scale randomized poverty-reduction trial, the Moving-to-Opportunity study.^6^ On the other hand, natural experimental studies are more realistic, as they can evaluate differences in changes in health status across socio-demographic groups over time after the implementation of certain policy reforms or significant macro-socioeconomic changes.^7^ An alternative approach may be micro-simulations. For example, Kristensen et al simulated racial differences in the effect of a soda tax policy on weight loss among children in the United States and found that the policy may be more effective for African Americans and Hispanics than for non-Hispanic Whites.^8^

Recent remarkable developments in behavioural science, providing novel insights into people’s decision-making frameworks, are also helpful for understanding the mechanisms of differential responses to social and political interventions according to levels of social stress.^9^ The new wave of behaviour science may also strengthen the capacity to design interventions to address health inequality. For example, health communication techniques could be evaluated in terms of their persuasiveness to various social groups by applying recent behavioural theories.^10^

The last recommendation of the WHO Commission is to strengthen governance among social organizations or the various players that can potentially contribute to policy reforms. This is important not only for organizations but also for researchers who seek to uncover better evidence regarding health disparities. Health researchers cannot directly handle poverty, education, or the work environment, the main targets of social epidemiologic interventions, without systemic support. Better governance and effective cross-disciplinary collaborations are required for the research community to be successful in its work.
